# Splenic flexure resection with Hugo™ RAS: Trocar placement, docking settings, and surgical strategy

**DOI:** 10.1111/codi.70242

**Published:** 2025-09-24

**Authors:** Filippo Carannante, Valentina Miacci, Guglielmo Niccolò Piozzi, Martina Marrelli, Marco Caricato, Jim S. Khan, Gabriella Teresa Capolupo

**Affiliations:** ^1^ UOC Chirurgia Colorettale Fondazione Policlinico Universitario Campus Bio‐Medico Rome Italy; ^2^ Università Campus Bio‐Medico Rome Italy; ^3^ Department of Colorectal Surgery Portsmouth Hospitals University NHS Trust Portsmouth UK; ^4^ University of Portsmouth Portsmouth UK

**Keywords:** colon cancer, colorectal surgery, HUGO RAS, oncological surgery, robotic surgery, splenic flexure

## Abstract

**Introduction:**

Robotic surgery has been widely adopted in colorectal surgery worldwide over the last decade, but it is still associated with significant cost. With the recent introduction of new robotic platforms, several studies reported the feasibility of different surgical procedures using newer robotic platforms. This study aims to present the first case series of colonic splenic flexure resection with the novel platform robotic‐assisted surgery (RAS) Hugo™. Tips for effective setup of the system and detailed configuration of tilt and docking angles are also provided.

**Methods:**

Three cases of colonic splenic flexure resection with Hugo RAS™ system are reported. OR setup and port placement allowing for a full robotic resection were used to perform vascular ligation, colonic splenic flexure mobilization, and side‐to‐side linear intracorporeal anastomosis, without the need for re‐docking.

**Results:**

Mean docking time was 11 (range 8–14) minutes and the median total operating time was 243 (range 180–350) minutes. Mean estimated blood loss was 116 (range 50–300) mL. No intraoperative complications or conversions were observed. One patient developed postoperative pneumonia.

**Conclusions:**

Total robotic splenic flexure resection with Hugo™ RAS system is feasible with good postoperative outcomes. Further studies are needed to confirm our results and to evaluate the surgical strategy and setup.

## INTRODUCTION

The adoption and implementation of minimally invasive surgery (MIS), after more than two decades, has greatly improved the treatment and the perioperative outcomes of patients with colorectal cancer [1]. MIS limits the size of surgical incisions, thereby reducing wound healing time, associated pain, risk of infection, and recovery time. The application of robotic surgical systems has been the next improvement in MIS, aiming to reduce errors by optimizing the surgeon's performance. Robotic platforms provide optimized and stabilized vision, improved dexterity through instrument articulation, improved dissection through motion scaling, and enhanced ergonomics [2]. Robotic surgery has been dominated by the da Vinci (Intuitive Surgical, Ltd., Sunnyvale, CA, USA) platforms since the early 2000s. Intuitive Surgical has developed the standard of robotic surgical platforms with three components (surgeon console, vision cart, and patient cart). Interestingly, the robotic surgical industry is growing rapidly, with various companies introducing innovative platforms to reduce operative costs [3]. The Hugo™ robotic‐assisted surgery (RAS; Medtronic, Minneapolis, MN, USA) platform is one of several alternatives to the da Vinci system [4]. This system consists of (1) system tower; (2) an open surgeon console, including a widescreen HD‐3D display with dedicated glasses, two pistol‐like handgrips for the control of the end‐effectors, and a footswitch panel; (3) four individual arm carts with a wide manoeuvre range. The Hugo™ RAS modular asset can be configured to support various surgical specialties such as urology, gynaecology, colorectal, and general surgery. To date, most of the available clinical reports in the literature have focused on gynaecological and urological surgical procedures [5, 6, 7], with very few studies describing its use in colorectal surgery [2, 4, 8, 9].

This study aims to describe the surgical strategy for performing robotic splenic flexure resection with the Hugo™ RAS system showing tips for effective set up of the system and detailed configuration of tilt and docking angles.

## MATERIALS AND METHODS

### Study population

This is a single‐centre prospective study evaluating all consecutive patients undergoing robotic splenic flexure resection with the Hugo™ RAS system for colon cancer from May to September 2024 at a tertiary referral centre (Colorectal Department of Fondazione Policlinico Universitario Campus Bio‐Medico, Rome, Italy). All patients provided informed consent for research studies.

Primary aim was to report the surgical strategy for robotic splenic flexure resection for colon cancer. Secondary aim was to describe perioperative outcomes.

Inclusion criteria were (1) colonic splenic flexure adenocarcinoma; (2) robotic approach; (3) elective setting; (4) curative surgery; (5) age above 18 years. Exclusion criteria are (1) palliative surgery; (2) emergency setting; (3) open or laparoscopic approach.

Clinical staging was performed via colonoscopy with biopsy and thoracic/abdominopelvic computed tomography (CT).

All patients were discussed at the MDT meeting for treatment strategy.

Pathological staging was provided according to the American Joint Committee on Cancer (AJCC) 8th edition staging system [10] during data review.

Complications were assessed according to Clavien‐Dindo's classification [11].

Adjuvant chemotherapy protocol followed international guidelines. All patients were followed up according to institutional or national guidelines.

### Surgical team training

Simulation training was performed by the surgical team in January 2024, aimed to achieve confidence with the Hugo™ RAS. Formal training was then completed by two console surgeons, one bedside assistant surgeon, and one scrub nurse on a human cadaver lab at the ORSI Academy in Ghent (Belgium) in April 2024. All procedures were performed by the same surgical team, which was comprised of high‐volume surgeons with long experience in both MIS and colorectal surgery but novice in robotic surgery.

### Operating theatre setup and HUGO™ RAS docking

Patients were positioned on the operating table supine, with the arms alongside the body and the legs open on stirrups. The patients were carefully secured with a dedicated soft foam pad to prevent sliding in the Trendelenburg position (10–15°) and the lateral right position (10–15°). After induction of pneumoperitoneum (11 mmHg), using a Veress needle at the Palmer's point, an 11 mm epigastric or supraumbilical port was inserted, and an explorative laparoscopy was performed using the 30° robotic camera.

**FIGURE 1:**
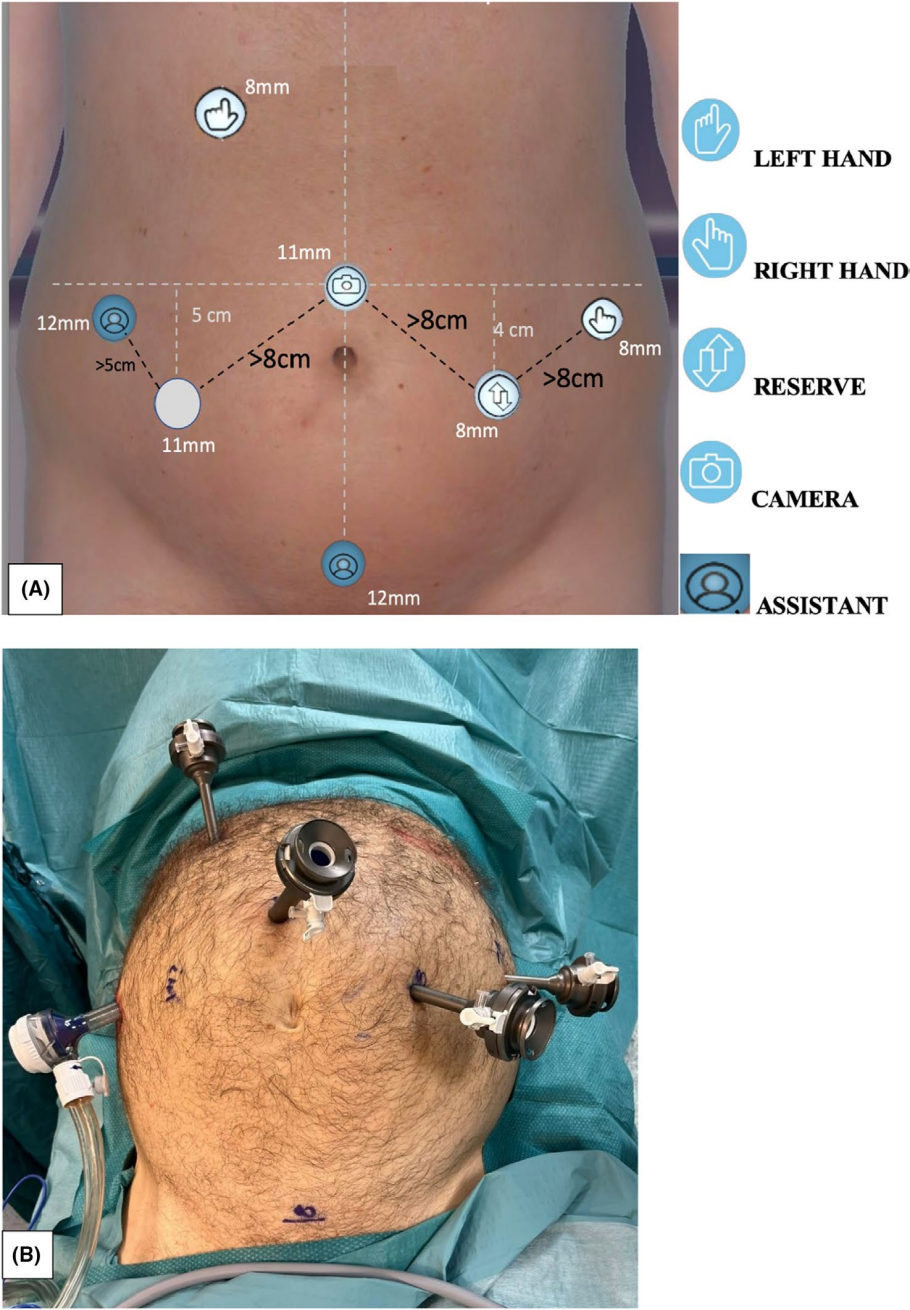
(A) Trocar layout for robotic splenic flexure colectomy. (B) Trocar placement in patient 1. The setup respects the minimum distance between the ports (8 cm). The accessory port is in the right flank. This placement was changed in the other two patients, locating the accessory port in the hypogastrium, on the line used for the extraction of the specimen, which allowed for greater assistance from the bed assistant.

**FIGURE 2:**
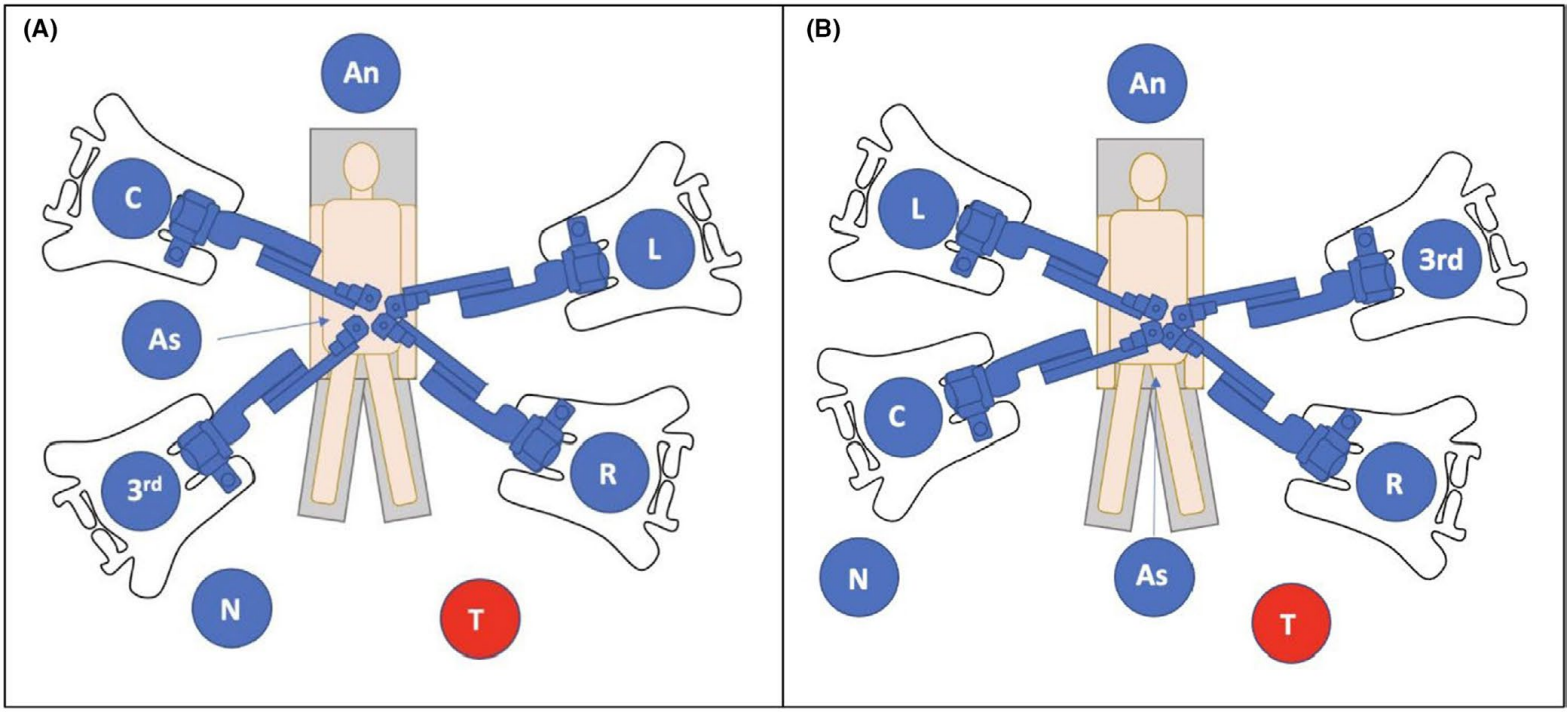
Operating room set up for the first case (A) and the second and third cases (B). 3rd, third arm; An, anaesthesiologist; As, bed assistant; C, camera; L, left hand; N, nurse; R, right hand; T, tower.

Three 8 mm robotic ports were placed after having identified the main body prominences (costal arches and antero‐superior iliac spines). The docking angles and the operating theatre setup were completed according to both the guidelines proposed by the manufacturer and to surgical experience. Two 8 mm ports were placed in the left flank, and the last 8 mm port was placed in the right hypochondrium. Each trocar was at least 8 cm apart from the others. An additional 11 mm port was placed for the bed assistant in the right flank, approximately 10 cm above the right iliac spine and 11 cm from the umbilical port in the first case. Due to the right tilt and the consequent mobilization of the small bowel, the team experienced that the assistant port in the right flank could inadvertently cause iatrogenic injury to the mesentery or small bowel during the instrument movements. For this reason, the assistant port positioning was changed in patient #2 and #3. An additional 11 mm port was placed in the suprapubic area for safety. The sites of the ports and the theatre setup are reported in Figures 1 and 2. Tilt and docking angles and other details of the procedures are reported in Table 1.

**TABLE 1:** Docking and tilt angles settings of the three cases.

	Instrument	Docking angle	Tilt angle
Patient 1			
Arm 1	Cadiere bipolar	43°	−15
Arm 2	Monopolar scissors	103°	+15
Arm 3	Cadiere forceps	203°	0
Arm 4	Camera	309°	+30
Patient 2			
Arm 1	Cadiere bipolar	56°	−15
Arm 2	Monopolar scissors	101°	+15
Arm 3	Cadiere forceps	247°	0
Arm 4	Camera	313°	+30
Patient 3			
Arm 1	Cadiere bipolar	52°	−15
Arm 2	Monopolar scissors	105°	+15
Arm 3	Cadiere forceps	245°	0
Arm 4	Camera	315°	+30

## RESULTS

### Patient characteristics

A total of three patients affected by colonic splenic flexure tumour underwent robotic splenic flexure resection with the HUGO™ RAS system. Characteristics of patients and primary tumours are listed in Table 2.

**TABLE 2:** Patient characteristic.

	Patient 1	Patient 2	Patient 3
Age, years	63	57	77
Sex	F	M	M
BMI, kg/m [2]	22.4	33.6	22.2
ASA score	3	3	2
Histology	Adenocarcinoma	Adenocarcinoma	Adenocarcinoma
Clinical TNM stage	1	2b	2a
Surgical procedure	Splenic flexure resection	Splenic flexure resection	Splenic flexure resection
Docking time, min	14	11	8
Console time, min	200	350	180
Total operating time, min	240	410	225
Intraoperative complications	No	No	No
Estimated blood loss, mL	50	200	100
Type of anastomose	Intracorporeal Side‐to‐side	Intracorporeal Side‐to‐side	Intracorporeal Side‐to‐side
Specimen extraction site	Sovrapubic (Pfannesteil)	Sovrapubic (Pfannesteil)	Sovrapubic (Pfannesteil)
Conversion	No	No	No
Length of stay, days	3	7	4
Time to first flatus, days	1	1	1
Time to first bowel movement, days	1	2	2
Postoperative complications	No	Pneumonia	No
Clavien‐Dindo	0	2	0
Specimen length, cm	25	22	23
Pathological stage	pT2N0M0	pT4aN0M0	pT3N0M0
Harvested lymph nodes	18	34	27
Positive lymph nodes	0	0	0

*Note*: Intraoperative, postoperative and pathological outcomes.

Abbreviations: ASA score, American Society of Anaesthesiologists score; BMI, body mass index.

### Operative outcomes

All procedures were performed fully robotically without complications. There were no system failures or conversions to laparoscopy or open approaches. Mean docking time was 11 (range 8–14) minutes. Mean console time was 243 (range 180–350) minutes (Table 2).

### Postoperative course

In two cases, the postoperative course was uneventful. One patient had postoperative pneumonia, treated successfully with antibiotics. Median postoperative hospital stay was 4.6 (range 3–7) days. Intraoperative, postoperative, and pathological results are reported in Table 2.

## DISCUSSION

This article reports the first three cases of splenic flexure resection for adenocarcinoma with the help of the Hugo™ RAS robotic platform. The use of robotic surgery has increased globally. The Hugo™ RAS System was launched in 2021, offering a modular alternative in surgical robotics [12]. This platform has been used primarily in urology and gynaecology, with noninferiority comparative studies (compared to da Vinci) already demonstrated in prostatectomy and nephrectomy series [13, 14]. Experience with this new system in general surgery, and especially in major colorectal surgery, is very limited, represented only by a few case reports and small case series. The first ones were Bianchi et al. [6], who reported a case series of three patients undergoing robotic‐assisted colectomies: one left colectomy and two right colectomies. In the same year, Gangemi et al. [15] reported on 17 patients undergoing robotic ileocecal resection, cholecystectomy, subtotal gastrectomy, sleeve gastrectomy, Nissen fundoplication, right hemicolectomy, and sigmoidectomy. Caruso et al. [16] described the first case of right hemicolectomy with the Hugo™ RAS system. Just a year later, Romero‐Marcos et al. [17] reported a case series of 10 patients undergoing right colectomy, sigmoid resection, high rectal resection, and ventral mesh rectopexy. Belyaev et al. [3], in June 2024, managed to publish a case series of 70 patients (55/70 underwent colorectal surgery), suggesting that most surgical interventions are feasible with no technical issues. More recently, Rottoli et al. [18] reported the largest series of 31 patients undergoing rectal resection with total mesorectal excision, and the same group published their robotic experience for inflammatory bowel disease [19] using the Hugo™ RAS system. To the best of our knowledge, so far, no experience has been reported regarding colonic splenic flexure resections with the Hugo™ RAS system. This study shows that the adoption of the Hugo™ RAS system for performing splenic flexure resection is feasible and safe. Global experience with splenic flexure cancer is limited because of its low incidence [20], and quite often extended resections are preferred to segmental resections. Nevertheless, segmental resection is a safe and effective treatment option for these cancers. Degiuli et al. [21], in their nationwide study of the Italian Society of Surgical Oncology‐Colorectal Cancer Network (SICO‐CNN), have proved that the minimally invasive approach met the criterion for noninferiority for postoperative complications and pathological outcomes, and have provided results of overall survival, cancer‐specific mortality, and response rate comparable to those of open resection.

The present study, with its limitation in numbers, shows improvement in the learning curve between cases with shorter docking time in general and shorter console and operating time between patient no. 3 and no. 1. Patient no. 2 was the most technically challenging due to the high BMI (33.6 kg/m^2^) and advanced tumour (T4a) causing an increase in operating time and estimated blood loss (200 mL). However, it was completed successfully without any intraoperative complication or need for conversion, showing that the Hugo™ RAS system is safe and feasible also for complex cases.

Robotic surgery provides invaluable assistance in performing splenic flexure colectomy thanks to magnified vision and articulated instruments that facilitate the performance of dissection and intracorporeal anastomosis [22]. The successful use over time of the Hugo™ RAS system in general surgery and more specifically in colorectal surgery is based on its open modular design allowing improved communication within the surgical team. This was experienced by our team where the open console setup and the absence of a voluminous single‐column robot allow for a linear communication between the assistant and the console surgeon. This is crucial especially since the console surgeon does not have direct control on energy devices and stapling, not currently integrated in the platform, which are under control of the assistant. Moreover, the flexibility in the configuration of the four modular arms allows the surgeon to have a more customized approach to surgical planning which can provide some freedom and similarity to laparoscopy since it does not require the linear port setup which is used with single‐column robots. The docking process is more articulated and demands training because all arms are attached to individual carts that must be placed in the correct position with an appropriate arm angulation as described in this report to limit clashes and optimize workflow. If these parameters are not respected, the optimal angles and arm movements will be compromised during the surgery. The ergonomic position of the console for the surgeon, the pistol‐like grips resembling laparoscopic instruments, and the lower cost have been discussed as further possible advantages of this robotic platform [23]. On the other hand, the system has some disadvantages since a complete range of staplers and advanced energy is not yet available and those devices, when needed, must be operated by the assisting surgeon through a conventional laparoscopic port [3]. Finally, the use of indocyanine green fluorescence (ICG) is not possible yet with this system. Many studies have confirmed the role of ICG to evaluate the perfusion of both bowel stumps used to create the anastomosis making it an essential tool in colorectal surgery [24]. This is true especially in splenic flexure resection, where congenital factors or acquired disease may result in interindividual variation in vessel branching patterns and dominant vessels, leading to reduced perfusion and increased risk of anastomotic leak [25].

This study has limitations. First, this is a limited series of three patients; therefore, the results should be confirmed by more extended series. However, it must be noted that despite a limited number of currently available platforms, the adoption of the Hugo™ RAS system is rapidly growing globally, with a consequent need for scientific support, especially for rare procedures such as splenic flexure colon cancer resections. Second, this study is limited to evaluating the perioperative outcomes but fails to provide a long follow‐up to evaluate the oncological outcomes.

This study has several strengths. This is the first series on robotic splenic flexure resection with the Hugo™ RAS with good outcomes. Despite the limited experience in robotic surgery of the surgeons, the docking time reduced through the cases.

## CONCLUSIONS

Full robotic splenic flexure resection with the use of the Hugo™ RAS system is feasible and safe and offers satisfactory perioperative outcomes. Further experience and clinical data are needed to standardize robotic colonic surgery techniques and to facilitate diffusion and integration of this new robotic system into robotic general surgery and colorectal programmes.

## AUTHOR CONTRIBUTIONS

**Filippo Carannante:** Conceptualization; methodology; formal analysis; investigation; writing – original draft. **Valentina Miacci:** Methodology; investigation; formal analysis; writing – original draft. **Guglielmo Niccolò Piozzi:** Writing – review and editing. **Martina Marrelli:** Investigation; formal analysis; data curation. **Marco Caricato:** Writing – review and editing. **Jim S. Khan:** Writing – review and editing. **Gabriella Teresa Capolupo:** Conceptualization.

## FUNDING INFORMATION

No funding was received for conducting this study.

## CONFLICT OF INTEREST STATEMENT

Jim S Khan performs proctoring for Intuitive Surgical and educational activity with Johnson & Johnson. Filippo Carannante, Valentina Miacci, Guglielmo Niccolò Piozzi, Martina Marrelli, Gianluca Costa, Marco Caricato, and Gabriella Teresa Capolupo have no conflict to disclose.

## ETHICS STATEMENT

We obtained the ethical statement approval in December 2022 from Fondazione Policlinico Universitario Campus Bio‐medico di Roma Committee (number: SC 2022.171).

## Data Availability

The data presented in this study are available on reasonable request from the corresponding author.
